# Hypovitaminosis A coupled to secondary bacterial infection in beef cattle

**DOI:** 10.1186/1746-6148-8-222

**Published:** 2012-11-14

**Authors:** Xiuyuan He, Yongtao Li, Meng Li, Guangmin Jia, Haiju Dong, Yanru Zhang, Cong He, Chuanqing Wang, Lixin Deng, Yurong Yang

**Affiliations:** 1 College of Animal Science and Veterinary Medicine, Henan Agricultural University, Wenhua Road 95#, Zhengzhou, Henan, 450002, P.R. China; 2 Unit of Animal Infectious Diseases, State Key Laboratory of Agricultural Microbiology, Huazhong Agricultural University, Shizishan Street1#, Hubei, Wuhan, 430070, P.R. China; 3 College of Veterinary Medicine, South China Agricultural University, Wushan Street483#, Guangdong, Guangzhou, 510642, P.R. China

**Keywords:** Vitamin A, Hypovitaminosis A, Calves, E. coli

## Abstract

**Background:**

Vitamin A is essential for normal growth, development, reproduction, cell proliferation, cell differentiation, immune function and vision. Hypovitaminosis A can lead to a series of pathological damage in animals. This report describes the case of hypovitaminosis A associated with secondary complications in calves.

**Case presentation:**

From February to March in 2011, 2-and 3-month old beef calves presented with decreased eyesight, apparent blindness and persistent diarrhea occurred in a cattle farm of Hubei province, China. Based on history inspection and clinical observation, we made a tentative diagnosis of hypovitaminosis A. The disease was confirmed as a congenital vitamin A deficiency by determination of the concentrations of vitamin A in serum and feed samples. Furthermore, pathological and microbiological examination showed that the disease was associated with pathogenic Escherichia coli (E. coli) infection and mucosal barriers damage in intestines. The corresponding treatments were taken immediately, and the disease was finally under control for a month.

**Conclusions:**

To our knowledge, this is the first report of hypovitaminosis A coupled to secondary infection of E. coli in beef cattle, advancing our knowledge of how vitamin A affects infection and immunity in animals. This study could also be contributed to scientific diagnosis and treatments of complex hypovitaminosis A in cattle.

## Background

Vitamin A is well known to be important for animal growth, development and various physiological processes, such as keeping the normal sight and bone function, especially in neonatal and growing animals [1]. Vitamin A is not synthesized by vertebrates and depends essentially onβ-carotene of green plants in dietary provision [2]. Since the discovery of vitamin A in 1920s, cases have been reported frequently about hypovitaminosis A in animals [3,4]. Many studies have showed that hypovitaminosis A can lead to a series of pathological damage, such as growth stunting, reproductive dysfunction, and low immunity, epithelial keratinization and degeneration, xerophthalmia and night blindness [5-7]. Researchers also demonstrated that vitamin A deficiency can be harmful for protein synthesis and maintenance of normal growth and metabolism, and decrease the body resistance to infection [8,9]. For grazing animals, vitamin A deficiency occurred easily in spring or winter when it lacks of green feed. To date, several investigations have been conducted on vitamin A deficiency in cattle [10-13]. In humans, there has been reported that vitamin A decreased during the acute phase response to bacterial infection [14]. However, the case of vitamin A deficiency in beef calves accompanied by bacterial infections has not yet been reported. In this study, we report on an investigation of vitamin A deficiency with E. coli infection in beef calves, a systematic diagnostic and effective treatment programs conducted on a beef cattle farm, which could be contributed to scientific prevention and control of vitamin A deficiency in cattle.

## Case presentation

### Case history

In Suizhou of Hubei Province, there is a beef cattle farm with a total of 553 cattle, including 144 cows, 66 pregnant cows, 59 calves and 284 finishing cattle. From February to March in 2011, the symptoms of elevated body temperature, loss of appetite and blurred vision were detected in 12 calves at the age of 2- to 3-month and 4 calves were total blind in April. The cows which produced calves last year could be found mainly lower abdomen edema with 10% morbidity, and 6 of 13 newborn calves appeared illness with 46% morbidity. The cattle diet included 0.5 to 1 kg concentrate feed (53% maize, 18% soybean meal and 29%wheat bran), 3.5 to 5 kg straw, and 15 kg (fresh weight) white lees, without any additives.

### Clinical signs

The earliest changes of all affected calves were anorexia, slow growth and weakness. Though clinical signs of affected calves varied, the common presence included mild to severe ataxia, partial blindness or total blindness as judged by avoidance of obstacles, rumen distention and persistent diarrhoea. Even in bright sunlight, some affected calves also lost light reflex. There was also absence of the blink response or eye preservation reflex. Compared to normal cattle, all affected calves had significantly increased defecography and urination. Examination of the eye revealed a dilated and circular pupil in affected calf, but horizontal and oval one in the normal.

### Ophtalmological examination

**Figure 1:**
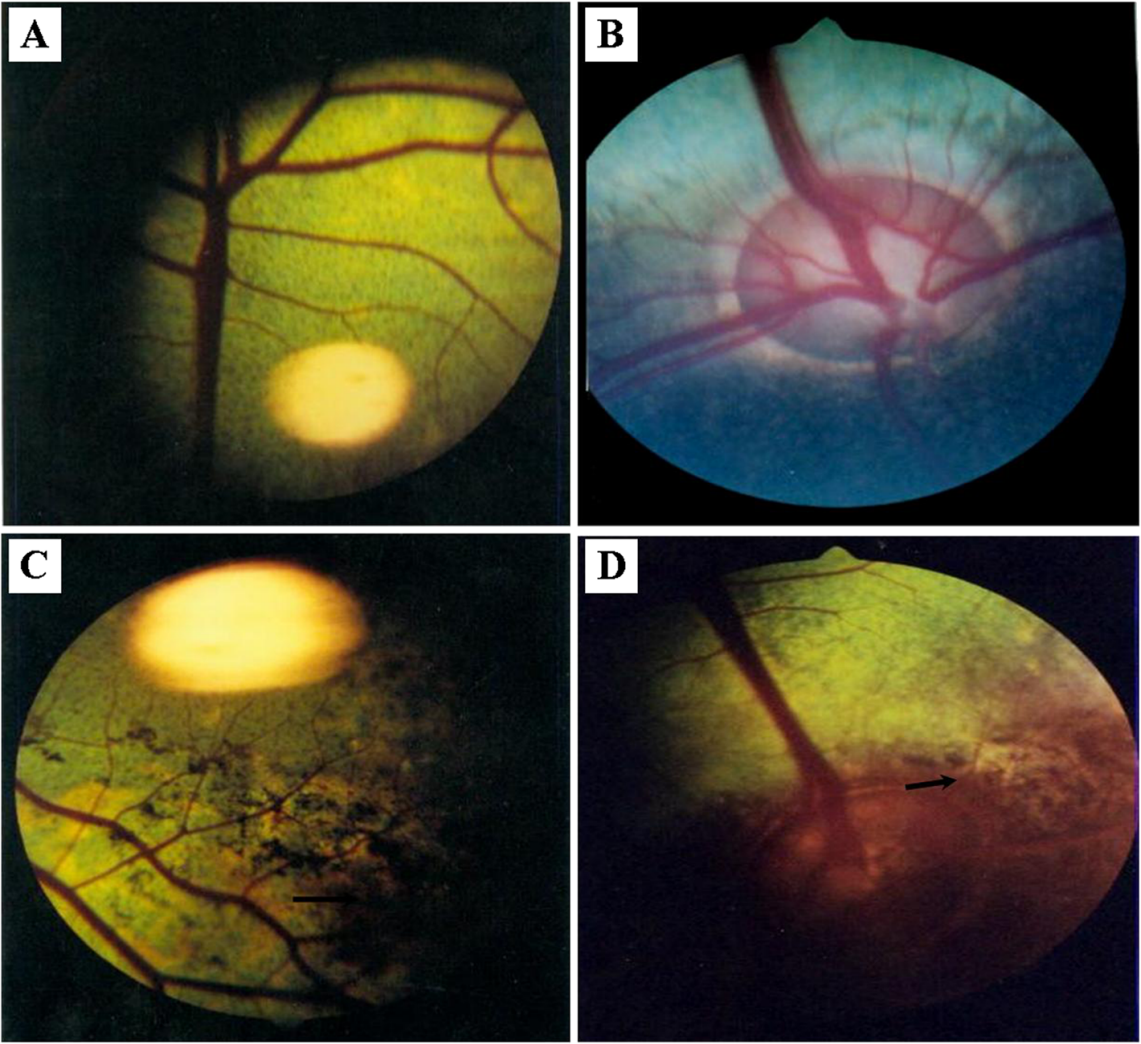
**Fundus examination of the healthy and affected calves.** In healthy calf, the surface of optic disc is flat, and the edge is clear and visible (Figure 1A). No edema, hemorrhage, exudation and disorder of pigment were found in retina (Figure 1B). The irregular pigmentation was present with varied sizes at local areas in the tapetum nigrum (Figure 1C) and tapetum lucidum (Figure 1D), and more obvious in the tapetum nigrum than in the tapetum lucidum. The black arrow in the figure 1C means that pigmentation was present in the tapetum nigrum and that in the figure 1D means that pigmentation was present tapetum lucidum.

The affected cattle were restrained in six columns for fundus examination and photography. The changes of optic disc, retinal vessels, tapetum nigrum and tapetum lacidum were carefully checked, and the healthy calves were also exampled as control. In healthy calf, the surface of optic disc was flat, and the edge was clear and visible (Figure 1A). No edema, hemorrhage, exudation and disorder of pigment were found in retina (Figure 1B). However, ophthalmoscopic examination of the affected calves revealed that irregular pigmentation was present with varied sizes at local areas in the tapetum nigrum(Figure 1C) and tapetum lucidum (Figure 1D), and more obvious in the tapetum nigrum than in the tapetum lucidum. No other significant abnormality was found.

### Determination of the content of vitamin A and aflatoxin B1

Through the history inspection and clinical signs, we suspected the disease might be related to vitamin A deficiency, then determined the vitamin A content using the High-performance liquid chromatography (HPLC) in milk and serum after sample preparation [15]. And we also randomly chose five pieces of feed samples and respectively measured the content of vitamin A according to national standard for measuring vitamin A in China. The limitation value of Vitamin A measured by this method in feed was 1000 IU/kg. Results showed that the average vitamin A content in the feed samples was far below the normal value (Table 1). Likewise, vitamin A was deficient in the milk of affected cows and in serums of affected calves [16]. In brief, we could conclude that the lack of Vitamin A in feed attributed to the low content in milk or serums (Table 1).

**Table 1:** Vitamin A concentration in different samples from clinically affected calves compared to reference values

**Samples**	**Concentrations**	**Normal value (16)**
Feed^a^	1360 IU/kg	400000 IU/kg
Milk^b^	36.1 IU/L	698 IU/L
Serum A^c^	1055 IU/L	43605 IU/L
Serum B^d^	130 IU/L	43605 IU/L

**a** Five feed samples were chose randomly and vitamin A concentration was shown in average (p < 0.01).

**b** Three milk samples from the affected cows were chose randomly and vitamin A concentration was shown in average(p < 0.05).

**c** Serum A was prepared from the affected calves at the early stage of disease.

**d** Serum B was prepared from the affected calves at the late stage.

Moreover, in the cattle farm of China, aflatoxin B1 is the most common aflatoxin and the necessary inspection item in feed. In order to verify whether the disease was related to aflatoxin poisoning, we also measured the aflatoxin B1 [17] and found that the concentration was 8.3 μg/kg in feed far less than the lowest poisoning dose of 100 μg/kg [18], indicating that the disease was not associated with aflatoxin B1 poisoning.

### Microbiological examination

To obtain the possible bacteria related to the diarrhea in diseased cattle, tissue samples including liver, spleen, lymph nodes, intestine and lungs were prepared and inoculated on blood agar for 18–24 h at 37°C. Rectal swabs were directly inoculated on blood agar. The suspected colonies were picked for smearing, staining and microscopic examination and inoculated onto eosin methylene blue agar medium and MacConkey medium for identification cultivation. Meanwhile, homogenates were inoculated in nutrient broth medium, and cultured for 24 hours in a carbon dioxide incubator with 5% carbon dioxide. Bacterial isolation and identification test showed that bacteria could only be isolated from and rectal swabs and the bacterial isolates were Gram-negative, rod-shaped, slightly rounded at both ends, without mobility by microscopic examination [19]. When grown on ordinary plate, the single colony was neat in edge, centrally uplifted, smooth, transparent and colourless. Furthermore, basic biochemical identification tests showed that the bacteria could ferment a variety of carbohydrates to produce acid and gas, especially ferment lactose rapidly and about half of the bacteria could not breakdown sucrose. MR (Methyl Red) and indole test were positive, the VP (Vogues Proskauer) and citrate experiments were negative, in accordance to the general characteristics of E. coli (Table 2), which further suggested that the isolates were E. coli. In addition, serotyping was performed on all isolates with 25 prevalent E. coli antiserums in China and confirmed the presence of O8 serotype E. coli in rectal swabs. To determine the virulence of these O8 strains, the fifty percent mouse lethal dose (MLD_50_) titer of no.1 strain was determined by intraperitoneally inoculating six mice in each group with 50 μl of serial 10-fold dilutions E. coli. Results indicated that this strain displayed high pathogenic to mice and LD_50_ was 5.45 × 10^9^ CFU (Table 3). For investigating the antibiotic susceptibility, the identified isolates were cultivated by streak plate method and the single colonies were picked for three continuous generations. The purified bacteria were inoculated on blood agar dish with 14 kinds of antibiotic discs incubated for 18 hours at 37°C. Results showed that the isolated strains are highly sensitive to amoxicillin, ciprofloxacin, gentamicin and other antibiotics (Table 4).

**Table 2:** Biochemical properties of the bacterial colonies isolated from intestine and rectal swabs of affected calves

**Test**	**Results**	**Test**	**Results**	**Test**	**Results**
Xylose	+	Galactose	+	Salicylic Acid	-
Glucose	+	Arabinose	+	Indole	+
Maltose	+	Cellobiose	-	Motility	-
Rhamnose	+	Fructose	+	Contact	+
Sorbitol	+	Lactose	+	Lysine decarboxylase	+
Manicol	+	Gelatin	-	Vogues Proskauer	-
Raffinose	-	Citrate	-	Methyl Red	+
Trehalose	+	Sucrose	-		

+indicates positive; -indicates negative.

**Table 3:** Pathological and microbiological findings from five affected calves

**Calf no.**	**Pathological changes**^**a**^	**E. coli serotype**^**b**^	**Positive samples**	**MLD**_**50**_**for E. coli (CFU)**^**c**^
1	severe	O8	intestine, rectal swabs	5.45 × 10^9^
2	moderate	O8	intestine	ND ^**d**^
3	severe	O8	intestine, rectal swabs	ND
4	severe	O8	intestine, rectal swabs	ND
5	mild	O8	intestine	ND

**a** The pathological changes of intestines from affected calves were estimated by visual examination.

**b** The serotypes of each strains were determined by agglutination test with 25 prevalent antiserums of pathogenic E. coli.

**c** MLD50 were determined by intraperitoneally inoculating six mice in each group with 50 μl of serial 10-fold dilutions E. coli and calculated by the method of Hamilton et al. [20].

**d** ND indicates that the LD_50_ of these strains was not determined.

**Table 4:** The antibiotic sensitivity test of E. coli strains isolated from affected calves

**Drugs**	Bacteriostatic diameters*	**Drugs**	**Bacteriostatic diameters**
amoxicillin	24	neomycin sulfate	10
cefoxitin	11	amikacin	15
ceftriaxone sodium	12	azithromycin	11
ceftiofur sodium	12	tylosin	12
vancomycin	11	doxycycline	8
streptomycin	8	florfenicol	12
gentamicin	20	ciprofloxacin	22

*: The diameters (d) ≥ 15 mm indicates highly sensitive; 10 mm ≤ d ≤ 15 mm shows moderately sensitive; d ≤ 10 mm indicates low-level sensitive.

### Pathological examination

**Figure 2:**
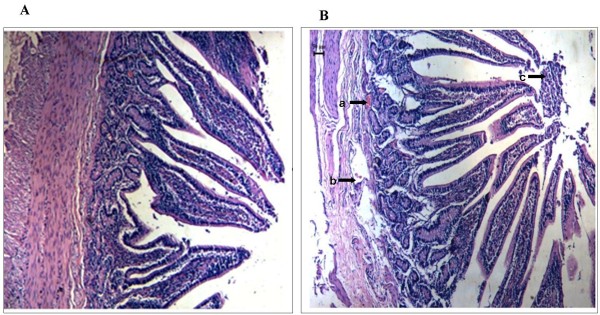
**The microscopic examination of jejunum in affected calves compared with the healthy jejunum.** (**A**) Hematoxylin- and eosin-stained sections of jejunum from the healthy calves. Images were obtained on an Olympus BX-50 light microscope at 5-fold original magnifications. The jejunum of the healthy calves showed normal epithelial cell in mucosa and submucosa. (**B**) The microscopic lesions of jejunum tissues collected from affected calves showed intestinal capillary congestion (arrow a), cell necrosis in smooth muscle (arrow b) and exfoliation of epithelial cells in mucosa (arrow c).

As mentioned above, it was intriguing to find that the affected calves manifested persistent diarrhoea. To exploit the pathological mechanism of diarrhoea, the intestines of healthy and affected calves were isolated and fixed by submerging them in 4% neutral buffered formalin and embedding them in paraffin. Three-micron sections were made before they were stained with hematoxylin and eosin (H&E). Images were obtained on an Olympus BX-50 light microscope at 5-fold original magnifications. Intestine tissues in lesion areas of affected calves showed intestinal capillary congestion, cell necrosis in smooth muscle and exfoliation of epithelial cells in mucosa (Figure 2A). Compared with the affected, the jejunum of the healthy calves showed normal epithelial cell in mucosa and submucosa (Figure 2B).

### Treatment

Considering the above clinical findings and experimental tests, we took the following measures to treat and control the disease. Firstly, to the affected calves, it is effective for controlling vitamin A deficiency through repeated intramuscular injections of vitamin A or continuous dietary supplementation. The affected cows and calves were intramuscularly administered by vitamins A for a month at the dose of 200000 IU once per day and 50000 IU respectively. Additionally, the severe affected cows and calf was orally treated respectively by 30 ml and 2 ml cod liver oil each day for a month. Secondly, antibacterial drugs, such as amoxicillin, ciprofloxacin and gentamicin were used to control secondary infection of E. coli. Last but not the least, calcium gluconate or glucose and mannitol were intravenously injected to alleviate cerebral edema and intracranial pressure, which contributes to reduce the neurological symptoms of the affected cattle. After one and a half months for treatment, most of mildly affected calves had a recovery of sight and the secondary E. coli infection was also effectively controlled. However, two calves with severe signs were totally blind in the end. In brief, the effect of corresponding treatments confirms also that the disease was attributed mainly to hypovitaminosis A.

## Discussion

Vitamin A of grazing animals is totally dependent on exogenous supply. According to previous studies, the minimum requirement of vitamin A in cow is about 30 IU/kg each day, and the demand should be increased by 50% during lactation and pregnancy [13]. The long-term lack of dietary vitamin A or carotene in cow can easily result in hypovitaminosis A in newborn calves [11]. The rapid growth and development of calves need enough vitamin A, through liver storage and extra implements, mainly dependent on breast milk. However, in our current study, beef cattle were fed with rice straw and distillers’ grains for a long time, without green feed, carrots and other vitamin A-rich feed, which resulted in vitamin A deficiency in pregnant and lactation cows. Under normal circumstances, the content of vitamin A in the cattle feed should not be less than 400,000 IU/kg in order to ensure normal growth and development. However, the vitamin A content was 1360 IU/kg in feed of this farm. In the serums of affected calves, the average content of vitamin A was 130 IU/L at the late stage in this farm, far below the normal value [16], confirming this disease was related with vitamin A deficiency in calves.

The blindness disorders are regarded as the typical signs in affected calves of vitamin A deficiency [21,22]. The mechanisms of blindness due to vitamin A deficiency have been explored for many years. In terms of blindness or associated signs, fundus examination plays an important role in the quick diagnosis of hypovitaminosis A. At the early stage of hypovitaminosis A, papilledema is the first sign of changes in the optic disc and is reversible under the experimental conditions. The most representative characteristic, however, is pigmentation with various sizes and shapes on the tapetum nigrum of retina. In this study, no clear papilledema was found, which might be due to the delay of taking photos of fundus. However, the irregular pigmentation was found clearly with varied sizes at local areas in the tapetum nigrum and tapetum lucidum, especially in tapetum nigrum (Figure 1).

Vitamin A plays a significant role in immune system function of animals. In humans, it is well accepted that `vitamin A deficiency impairs innate immunity by impeding normal regeneration of mucosal barriers damaged by infection, and by diminishing the function of immune cells [23,24]. In the case of diarrheal diseases, vitamin A could promote regeneration of damaged mucosal epithelium and enhance the phagocytic activity of neutrophils and macrophages. It has also been shown that vitamin A can reduce the incidence and duration of diarrhea in children [25,26]. In this study, based on the clinical findings, his-pathological changes in intestines and vitamin A determinations, we could conclude that hypovitaminosis A impeded mucosal barriers of intestines and lowered the immunity of cattle which made the cattle more susceptible to E. coli, and the intractable diarrhea of cattle was likely attributed to both hypovitaminosis A and pathogenic E. coli infections, which was in accordance to previous studies [27,28]. Therefore, the corresponding treatment should include vitamin A supplement and antibacterial drugs utilization according the antibiotic susceptibility test. It is intriguing that Rumen distention was present in affected calves, whether it is due to hypovitaminosis A or E. coli infections remains further studies. Further research on E. coli isolates should be performed to identify genetic material encoding for specific virulence factors of E. coli and to demonstrate the effect of vitamin A deficiency on expression of innate immunity-related genes and development of immune cells in gastrointestinal tract of cattle [29].

## Conclusions

This report describes a predominant case about hypovitaminosis A coupled to secondary E. coli infection in beef calves. Based on the clinical signs and experimental tests, corresponding measures were taken immediately to make vitamin A recovery at the normal level and to control E. coli infection in intestines of calves. Collectively, the findings provided insights into the important role of vitamin A in the immunity against microbiology infections of animals.

### Consent

Orally informed consent was obtained from the owner of cattle farm for publication of this case report and any accompanying images.

## Abbreviations

(VA): Vitamin A; (E. coli): Escherichia coli; (HPLC): High-Performance Liquid Chromatography; (MR): Methyl Red; (MLD_50_): Fifty percent mouse lethal dose.

## Competing interests

There are non-financial competing interests in this study.

## Authors’ contributions

XYH and YTL carried out all works and drafted the manuscript. ML, GMJ, HJD and LXD performed the clinical examination and the course of the case. YRY and YRZ performed the pathological examination. CH and CQW participated in microbiological test in the lab and helped to draft the manuscript. All authors read and approved the final manuscript.
